# Caregivers’ provision of sweetened fruit-flavoured drinks to young children: importance of perceived product attributes and differences by socio-demographic and behavioural characteristics

**DOI:** 10.1017/S1368980022000751

**Published:** 2022-08

**Authors:** Yoon Y Choi, Melissa L Jensen, Frances Fleming-Milici, Jennifer L Harris

**Affiliations:** 1 Rudd Center for Food Policy and Obesity, University of Connecticut, Hartford, CT 06103, USA; 2 Korea Rural Economic Institute, Naju-Si, Jeollanam-do 58321, Republic of Korea

**Keywords:** Sugar-sweetened beverages, package claims, public policy, parent attitudes, nutrition knowledge

## Abstract

**Objective:**

Drinks containing added sugar and/or non-nutritive sweeteners are not recommended for children under 6 years. Yet, most young children consume these products. The current study examined factors associated with caregivers’ provision of sweetened drinks to their young child.

**Design:**

Caregivers reported frequency of providing sweetened fruit-flavoured drinks (fruit drinks and flavoured water) and unsweetened juices (100 % juice and juice/water blends) to their 1- to 5-year-old child in the past month and perceived importance of product attributes (healthfulness, product claims and other characteristics), other drinks provided, reading the nutrition facts panel and socio-demographic characteristics. A partial proportional odds model measured the relationship between these factors and frequency of providing sweetened fruit-flavoured drinks.

**Setting:**

Online cross-sectional survey.

**Participants:**

U.S. caregivers (*n* 1763) with a young child (ages 1–5).

**Results:**

The majority (74 %) of caregivers provided sweetened fruit-flavoured drinks to their child in the past month; 26 % provided them daily. Provision frequency was positively associated with some drink attributes, including perceived healthfulness, vitamin C claims and box/pouch packaging; child requests and serving other sweetened drinks and juice/water blends. Provision frequency was negatively associated with perceived importance of ‘no/less sugar’ and ‘all natural’ claims. Reading nutrition facts panels, serving water to their child and child’s age were not significant.

**Conclusion:**

Misunderstanding of product healthfulness and other marketing attributes contribute to frequent provision of sweetened drinks to young children. Public health efforts to address common misperceptions, including counter marketing, may raise awareness among caregivers about the harms of providing sweetened drinks to young children.

Sugar-sweetened beverage (SSB) consumption in early childhood (birth to 5 years) contributes to long-term health risks, including overweight and obesity, dental caries, CVD and type 2 diabetes^(1–3)^. Drinking SSB more than three times a week is associated with childhood overweight or obesity^(4)^. Popular children’s fruit drinks and flavoured waters (i.e. sweetened ‘water beverages’) contain high amounts of sugar (median = 16 g/serving and 10 g, respectively). Moreover, 74 % of these drinks contain non-nutritive sweeteners, including 38 % that contain both non-nutritive and added sugar^(5)^. Consumption of drinks with non-nutritive sweeteners also raises concerns due to their potential long-term effects on children’s sweet preferences and a lack of high-quality research to ascertain their safety with young children^(6,7)^. Therefore, nutrition and health experts do not recommend serving these drinks to children under 6 years of age^(3,8)^. The American Academy of Pediatrics suggests a limited amount of unsweetened juice, including 100 % juice or juice/water blends (i.e. 100 % juice diluted with water and no added sweeteners) as a healthier alternative^(9)^. From 2006 to 2017, fruit drink volume purchases by households with young children (1–5 years) have declined, but they remained three times greater than unsweetened juice purchases. At the same time, households’ purchases of sweetened flavoured waters increased.

Young children’s home food environments are important determinants of obesity risk^(10)^, and caregivers’ misperceptions about the healthfulness of the food and drinks they serve their children may be significant contributors. A few studies have examined caregivers’ perceptions of different types of SSB and found that many believe that sweetened fruit-flavoured drinks are healthy choices for their child(ren)^(11,12)^. Caregivers’ misperceptions about the healthfulness of SSB were associated with serving them to their 2- to 17-year-old children^(11)^, and misperceptions of SSB healthfulness among Hispanic caregivers were associated with a greater number of SSB types served^(13)^. Furthermore, many caregivers rely upon on-package ingredient claims (e.g. ‘vitamin C,’ ‘low-sugar’) in their decision to purchase fruit drinks for their children, which may bias caregivers’ awareness of nutritional quality^(11,14)^. Educating parents about healthier alternatives to SSB, such as plain water and 100 % juice^(2)^, and greater use of nutrition facts panels^(15)^ have also been suggested to reduce children’s SSB consumption. Understanding additional behavioural and perceptual factors that might be associated with sweetened drink provision by caregivers would also inform effective interventions and policy solutions.

The objective of the current study is to investigate contributors to frequency of caregivers providing sweetened fruit-flavoured drinks (fruit drinks and flavoured water) to their 1- to 5-year-old children. The study examines (i) caregivers’ perceptions about healthfulness and other attributes of drink products, (ii) behavioural factors, including frequency of other drink type provision and usage of nutrition facts panels and (iii) socio-demographic factors.

## Methods

A cross-sectional online survey of caregivers conducted in October 2019 assessed reported frequency of providing sweetened fruit-flavoured drinks (fruit drinks and/or flavoured water) to their young child and reported reasons for providing them, including product attributes (perceived healthfulness, ingredients and other characteristics), other drinks provided, reading the nutrition facts panel and socio-demographic characteristics. All analyses were performed with Stata statistical software version 16 (Stata Corp).

### Procedures

A national online survey panel company (Innovate MR) recruited a random sample of caregivers with a young child (ages 1–5), with additional quota sampling to obtain 200 each Asian-Pacific Islander, Black and Hispanic caregivers for comparison purposes. Innovate maintains a large panel of members who agree to participate in online surveys^(16)^. Panel members are rewarded for their participation in the form of virtual currency that can be redeemed for gift cards, PayPal or charitable donations, but participants do not receive compensation for completing individual surveys. Participation in individual surveys is voluntary. Those who agreed to participate received an email link to the 25-minute Qualtrics survey. Survey respondents were first screened to identify those who had (i) at least one child between 1 year and 5 years old living at home, (ii) some responsibility for deciding what their children eat or drink and (iii) no children with a disease or condition that requires a special diet. If caregivers had more than one 1- to 5-year-old child, they were asked to answer questions about the child with the most recent birthday. Responses were collected in October 2019 and analysed in March 2021.

### Measures

The survey was composed of four sections: (i) frequency of providing different types of drinks to their child; (ii) reasons for serving them and other behavioural factors; (iii) familiarity and understanding of ingredients in popular children’s drinks and (iv) demographic characteristics. Results for section (iii) are published elsewhere^(17,18)^. Caregivers first reported whether they had provided any of the following drink types to their child in the past month: 100 % juice, juice/water blends, fruit drinks, flavoured water, plain water (bottle and tap), sweetened milk drinks (Nido, flavoured milk, Pediasure, toddler milk and smoothie drinks) and other types of drinks (nectar juices, sports drinks, regular soda, diet soda and iced teas). Within each drink type (100 % juice, juice/water blends, fruit drinks and flavoured water), caregivers selected the specific products (including brand and variety) they had provided, with options to indicate another brand/product provided or that they did not provide the drink type in the past month. They also indicated how often they gave their child the drink type in the past month (1 = less than once a week, 2 = about once a week, 3 = a few times a week, 4 = about once a day and 5 = more than once a day).

Caregivers then indicated how healthy they believed different drink types were (100 % juice, juice/water blends, fruit drinks and flavoured water) using a scale from 1 = very unhealthy to 10 = very healthy. They selected how often they look at the nutrition facts panel on the back or side of the package when they decide what drinks to buy for their child in the store (1 = never, 2 = only the first time, 3 = sometimes, 4 = most of the time and 5 = all the time). If they purchased any of these drink types, they also rated how important different product features were in their purchase decision (1 = not at all important to 7 = extremely important). Product features assessed included package claims (‘No added sugar’, ‘Less sugar’, ‘100 % juice’, ‘Good source of vitamin C’, ‘All natural’, ‘No diet sweeteners’, ‘Low in calories’, ‘Organic’, ‘No high fructose corn syrup (HFCS)’, ‘No artificial ingredients’ and ‘Non-GMO’), package type (i.e. juice box or pouch), their child asks for it and low price. These features were selected based on previous research on reasons caregivers purchase drinks for their children^(11)^ and common claims on drink product packages^(5)^. Caregivers also provided demographic information including age and gender of their child and caregiver education level, race/ethnicity, age and gender.

### Statistical analysis

Pairwise comparisons with Tukey HSD post hoc test examined mean differences in caregivers’ perceptions of drink types, including healthfulness of sweetened drinks and perceived importance of different product features, by age of child and caregivers’ education and race/ethnicity. Differences in caregivers’ behavioural factors included looking at the nutrition facts panel and serving different types of drinks were also examined by socio-demographic characteristic using pairwise comparisons of proportions with Bonferroni correction.

A partial proportional odds model identified factors associated with frequency of providing sweetened fruit drinks or flavoured water to their child as an ordinal dependent variable. The partial proportional odds model allows for a relaxed assumption of the underlying ordinal logistic regression parallel regression assumption^(19)^, as this assumption was violated (Brant test *χ*^2^ = 100·69, *P* < 0·001).

The dependent variable indicated the frequency of providing either fruit drinks or flavoured water in the past month (i.e. the frequency of the sweetened drink type provided most often) coded as (1) sweetened drinks not provided (only unsweetened drinks provided); (2) low (provided once a week or less); (3) moderate (provided a few times a week) and (4) high (provided once a day or more). Three models were estimated: never *v*. low-to-high, never/low *v*. moderate/high and never-to-moderate *v*. high.

Independent variables in the model included importance of product features in drink purchase decisions, perceived healthfulness of different drink types, provision of other drink types and demographic variables. Due to high correlation between some product feature responses (Pearson’s R > 0·5), two scales were created: natural ingredients (‘All natural’, ‘Organic’, ‘No HFCS’, ‘No artificial ingredients’ and ‘Non-GMO’, Cronbach’s Alpha = 0·9) and no/less added sugar (‘No added sugar’ and ‘Less sugar’, Cronbach’s Alpha = 0·8). Caregivers’ perceptions of the healthfulness of fruit drinks and flavoured water were averaged for the perceived healthfulness of sweetened drinks variable. Dichotomous variables were created for frequency of looking at nutrition facts panel (1 = most or all the time, 0 = never, only the first time I buy it, or sometimes) and frequency of plain water provision (1 = more than once a day, 0 = once a day or less). Provision of sweetened milk drinks, other drink types (nectar juices, sports drinks, regular soda, diet soda and iced teas), 100 % juice and juice/water blends were also coded as dichotomous variables (1 = provided in the past month, 0 = did not provide in the past month). Demographic variables included child’s age as a continuous variable and caregiver education and race/ethnicity as categorical variables.

## Results

Of 2591 total survey responses, 1829 agreed to participate and met all screening criteria. An additional 158 did not report frequency of providing sweetened drinks, for a 91 % completion rate (*n* 1671). Average child age was 3·1 years, and caregivers were diverse in race, ethnicity and education (see Table 1). About three-quarters (73 %) of participants reported that they had provided sweetened fruit drinks and/or flavoured water to their child in the past month; 39 % reported providing both. Frequency of providing sweetened drinks was approximately equally divided between never, low, moderate and high. In addition, more than 90 % of caregivers reported providing their child 100 % juice, 61 % provided juice/water blends and more than one-half provided sweetened milk drinks and other drinks (i.e. soda, diet soda and other SSB) in the past month. Almost 80 % of caregivers reported that they gave their child plain water at least once a day. Approximately one-half of caregivers reported looking at the nutrition facts panel most or all the time when deciding what drinks to purchase for their child. Perceived healthfulness of the sweetened fruit-flavoured and unsweetened juice drinks they chose for their child was lowest for fruit drink (M = 4·3 out of 10) and higher for 100 % juice (M = 7·8). The most important features of the drinks provided included 100 % juice, good source of vitamin C, no/less sugar and no diet sweeteners (M ≥ 5·1 of 7), while the least important features included low energies and comes in a box/pouch (M ≤ 3·9).

**Table 1:** Summary statistics (*n* 1671)

	*n*	Percent	Mean	sd
Age of 1- to 5-year-old child with the most recent birthday	–	–	3·1	1·4
Caregiver’s education				
Less than high school or high school/GED	351	22	–	–
Some college or 2-year college degree	658	41	–	–
4-year college degree, professional degree or graduate school	591	37	–	–
Race/ethnicity				
Non-Hispanic White	773	50	–	–
Non-Hispanic Black	208	14	–	–
Hispanic	317	21	–	–
Non-Hispanic Asian	130	8	–	–
Non-Hispanic Other	104	7	–	–
Drinks served to their child in the past month				
Both fruit drink and flavoured water	656	39	–	–
Fruit drink only	403	24	–	–
Flavoured water only	173	10	–	–
Unsweetened juices (100 % juice and/or juice/water blends) only	439	26	–	–
Freq of providing sweetened fruit drinks or flavoured water in the past month				
Never provided	439	26	–	–
Low (once a week or less)	420	25	–	–
Moderate (a few times a week)	370	22	–	–
High (once a day or more)	442	26	–	–
Provided other drinks in the past month				
100 % juice	1600	91	–	–
Juice/water blends	1072	61	–	–
Sweetened milk drinks	946	54	–	–
Other sweetened drinks (soda, diet soda and other SSB)	955	54	–	–
Freq of providing plain water in the past month				
Never provided	46	3	–	–
Once a week or less	78	5	–	–
A few times a week	241	14	–	–
Once a day	356	21	–	–
More than once a day	977	58	–	–
Freq of looking at nutrition facts panel				
Never	135	8	–	–
Only the first time I buy it	218	13	–	–
Sometimes	491	30	–	–
Most of the time	438	27	–	–
All the time	347	21	–	–
Perceived healthfulness of drink (1 = very unhealthy, …, 10 = very healthy)				
100 % juice	–	–	7·8	2·3
Juice/water blends	–	–	6·2	2·4
Flavoured water	–	–	5·7	2·7
Fruit drink	–	–	4·3	2·6
Perceived importance of product features (1 = not at all important, …, 7 = extremely important)				
100 % juice	–	–	5·4	1·5
Good source of vitamin C	–	–	5·2	1·5
No/less sugar	–	–	5·1	1·4
No diet sweeteners	–	–	5·1	1·9
Low price	–	–	4·8	1·7
Natural (all natural, organic, no HFCS, no artificial ingredients, non-GMO)	–	–	4·7	1·5
My child asks for it	–	–	4·7	1·7
Low in calories	–	–	3·9	1·9
Comes in juice box or pouch	–	–	3·8	2·0

SSB, sugar-sweetened beverage; HFCS, high fructose corn syrup.

Table 2 shows differences in caregivers’ perceptions of drink type healthfulness and importance of product features by socio-demographic characteristics. Caregivers with a 5-year-old child rated sweetened drinks as significantly healthier than caregivers with a younger child. They also rated box/pouch packages, my child asks for it and low price as significantly more important. Caregivers who completed 4 years of college or more also rated sweetened drinks as healthier and most product features as more important, compared with those with less education. Differences varied by race/ethnicity. However, Hispanic, non-Hispanic Black and non-Hispanic Asian caregivers rated several product features as significantly more important compared with non-Hispanic White caregivers.

**Table 2:** Differences in caregivers’ perceptions by socio-demographic characteristics*

		Perceived healthfulness of sweetened drinks†		Perceived importance of product features‡																	
				Natural§		100 % juice		Good source of vitamin C		No/less sugar		Low in calories		Comes in juice box or pouch		My child asks for it		Low price		No diet sweeteners	
		M	sd	M	sd	M	sd	M	sd	M	sd	M	sd	M	sd	M	sd	M	sd	M	sd
Age of child	1 year (*n* 269)^a^	3·5	2·0	4·8	1·5	5·5	1·5	5·5	1·4	5·3	1·4	4·1	1·9	3·5	2·1	4·4	1·8	4·6	1·7	4·9	2·0
	2 years (*n* 405)^b^	3·4	1·6	4·7	1·5	5·4	1·5	5·2	1·4	5·0	1·5	3·7	1·9	3·7	1·9	4·6	1·7	4·5	1·7	5·1	1·8
	3 years (*n* 393)^c^	3·6	0·7	4·7	1·5	5·4	1·5	5·2	1·4	5·1	1·4	3·9	1·9	3·8	1·9	**4·8**	1·6^a^	**4·9**	1·5^b^	5·2	1·8
	4 years (*n* 330)^d^	3·5	0·6	4·6	1·5	5·3	1·6	5·1	1·7	5·0	1·5	3·8	1·9	3·8	1·9	4·6	1·7	4·7	1·8	5·0	1·9
	5 years (*n* 366)^e^	**4·0**	2·0^a,b,c,d^	4·8	1·4	5·4	1·6	5·2	1·5	5·1	1·5	4·1	1·9	**4·2**	1·9^a,b,c,d^	**4·8**	1·6^a^	**5·0**	1·6^a,b,d^	5·1	1·8
Caregiver Education	≤ high school (*n* 351)^a^	3·7	1·6	4·4	1·5	5·2	1·6	5·2	1·6	4·8	1·5	3·8	1·9	3·7	2·0	4·6	1·7	4·9	1·7	4·8	2·0
	≤ 2-year college (*n* 658)^b^	3·4	1·6	4·6	1·5	5·3	1·5	5·2	1·4	5·0	1·4	3·8	1·9	3·7	2·0	4·6	1·7	4·8	1·6	5·1	1·8
	≥ 4-year college (*n* 591)^c^	**3·8**	2·2^b^	**5·0**	1·4^a,b^	**5·6**	1·4^a,b^	5·3	1·5	**5·3**	1·3^a,b^	**4·2**	1·8^a,b^	**4·1**	1·9^a,b^	4·8	1·6	4·7	1·7	**5·3**	1·8^a,b^
Race/ Ethnicity	Hispanic (*n* 317)^a^	3·7	1·8	**4·9**	1·4^e^	5·3	1·5	**5·4**	1·4^e^	5·1	1·4	**4·2**	1·7^e^	3·9	1·9	4·7	1·7	4·6	1·7	5·1	1·9
	Non-Hispanic Asian (*n* 130)^b^	3·2	1·7	**5·2**	1·4^e^	5·6	1·4	5·4	1·4	**5·4**	1·4^e^	**4·3**	1·8^e^	**4·3**	1·9^e^	4·4	1·8	4·7	1·6	5·5	1·6
	Non-Hispanic Black (*n* 208)^c^	3·7	1·9	**5·0**	1·3^e^	5·6	1·5	**5·5**	1·6^e^	5·2	1·4	**4·3**	2·0^e^	4·1	2·0	4·6	1·8	4·8	1·9	5·1	1·8
	Non-Hispanic other (*n* 104)^d^	3·5	1·4	4·7	1·7	5·4	1·6	5·2	1·6	5·0	1·5	3·7	2·0	3·8	2·0	4·8	1·7	5·1	1·7	5·0	2·0
	Non-Hispanic White (*n* 773)^e^	3·6	1·8	4·5	1·5	5·4	1·5	5·1	1·5	5·0	1·4	3·7	1·9	3·7	1·9	4·7	1·6	4·8	1·6	5·1	1·9

* Pairwise comparisons with Tukey post-hoc tests. Letter means that it is statistically different from the row of the letter at 5 % significance level.

† Sweetened drinks include fruit drink and flavoured water. 1 = very unhealthy, …, 10 = very healthy.

‡ 1 = not at all important, …, 7 = extremely important.

§ Natural feature includes ‘All natural’, ‘Organic’, ‘No HFCS’, ‘No artificial ingredients’ and ‘Non-GMO’.

Table 3 shows differences in behavioural factors by socio-demographic characteristics. The proportion of caregivers looking at the nutrition facts panel most or all the time was higher for caregivers with at least a high school diploma/GED and for non-Hispanic Asian caregivers. The proportion of caregivers who served soda, diet soda and other SSB in the past month was higher for caregivers with a 3- to 5-year-old child and those with less a than 4-year college degree. In addition, the proportion of caregivers who served 100 % juice in the past month was higher for those with a high school degree or less and for non-Hispanic Black caregivers.

**Table 3:** Differences in caregivers’ behavioural factors by socio-demographic characteristics*

		Looking at nutrition fact panel most or all the time	Served 100 % juice in the past month	Served juice/water blends in the past month	Served sweetened milk drinks in the past month	Served other sweetened drinks in the past month	Served plain water more than once a day in the past month
Age of child with the most recent birthday	1 year (*n* 269)^a^	51 %	86 %	57 %	52 %	41 %	48 %
	2 years (*n* 405)^b^	46 %	91 %	59 %	51 %	49 %	52 %
	3 years (*n* 393)^c^	48 %	93 %^a^	67 %	61 %	61 %^a,b^	49 %
	4 years (*n* 330)^d^	48 %	90 %	58 %	53 %	58 %^a^	53 %
	5 years (*n* 366)^e^	50 %	92 %	62 %	60 %	70 %^a,b,d^	61 %^a,c^
Caregiver’s education	≤ high school (*n* 351)^a^	35 %	94 %^b,c^	60 %	58 %	60 %^c^	53 %
	≤ 2-year college (*n* 658)^b^	45 %^a^	92 %	62 %	55 %	60 %^c^	50 %
	≥ 4-year college (*n* 591)^c^	61 %^a,b^	87 %	61 %	57 %	52 %	55 %
Race/ethnicity	Hispanic (*n* 317)^a^	49 %	94 %	70 %^e^	62 %	63 %^b,c,e^	52 %
	Non-Hispanic Asian (*n* 130)^b^	59 %^e^	87 %	57 %	53 %	41 %	42 %
	Non-Hispanic Black (*n* 208)^c^	55 %	97 %^b,e^	70 %^e^	56 %	57 %	48 %
	Non-Hispanic other (*n* 104)^d^	51 %	91 %	63 %	59 %	59 %	52 %
	Non-Hispanic White (*n* 773)^e^	44 %	88 %	56 %	55 %	56 %	57 %^b^

* Pairwise comparisons with Bonferroni corrections. Letter means that it is statistically different from the row of the letter at 5 % significance level.

Table 4 reports odds ratios from estimations of the partial proportional odds model (see Supplemental Table 1 for computed marginal effects of predictors). Higher perceived healthfulness of sweetened drinks and perceived importance of six of the nine product features were associated with likelihood of providing sweetened drinks to their child (*v* never providing them). Good source of vitamin C, child asks for it and package type were positively associated with providing sweetened drinks, while 100 % juice, no/less sugar and natural product features were negatively associated with frequency of provision of sweetened drinks. Product features that were not associated with provision of sweetened drinks included no diet sweeteners, low in energies and low price.

**Table 4:** Regression results with odds ratios

Frequency of providing sweetened drinks to a child	Never *v.* low to high		Never and low *v*. moderate and high		Never to moderate *v*. high	
	OR	95 % CI	OR	95 % CI	OR	95 % CI
Perceived healthfulness of sweetened drinks	1·56***	1·40, 1·74	1·58***	1·45, 1·73	1·35***	1·25, 1·46
Importance of product features						
100 % juice	0·83**	0·74, 0·93	0·97	0·87, 1·07	1·01	0·91, 1·12
Good source of vitamin C	1·10*	1·01, 1·19	1·10*	1·01, 1·19	1·10*	1·01, 1·19
No/less sugar	0·88*	0·80, 0·98	0·88*	0·80, 0·98	0·88*	0·80, 0·98
No diet sweeteners	0·97	0·91, 1·03	0·97	0·91, 1·03	0·97	0·91, 1·03)
Low price	1·04	0·98, 1·11	1·04	0·98, 1·11	1·04	0·98, 1·11)
Natural†	0·87*	0·78, 0·98	0·87*	0·78, 0·98	0·87*	0·78, 0·98
My child asks for it	1·11**	1·04, 1·19	1·11**	1·04, 1·19	1·11**	1·04, 1·19
Low in energies	1·05	0·99, 1·12	1·05	0·99, 1·12	1·05	0·99, 1·12
Comes in juice box or pouch	1·06*	1·00, 1·12	1·06*	1·00, 1·12	1·06*	1·00, 1·12
Freq of looking at nutrition fact panel ref=never-sometimes)						
Most of the time or all the time	1·18	0·95, 1·47	1·18	0·95, 1·47)	1·18	0·95, 1·47
Served unsweetened drink(s) in the past month (ref=no)						
100 % juice	1·26	0·81, 1·95	1·26	0·81, 1·95	1·26	0·81, 1·95
Juice/water blends	1·47***	1·19, 1·82	1·47***	1·19, 1·82	1·47***	1·19, 1·82
Served other sweetened drinks in the past month (ref=no)						
Sweetened milk drinks	1·79***	1·47, 2·19	1·79***	1·47, 2·19	1·79***	1·47, 2·19
Soda, diet soda and other SSB	2·75***	2·08, 3·62	1·67***	1·31, 2·12	1·39*	1·06, 1·83
Served plain water in the past month (ref=never-once a day)						
More than once a day	0·93	0·76, 1·13	0·93	0·76, 1·13	0·93	0·76, 1·13
Age of 1- to 5-year-old child with the most recent birthday	1·07	0·99, 1·15	1·07	0·99, 1·15	1·07	0·99, 1·15
Caregiver’s education (ref= ≤ HS)						
≤ 2-year college	0·78*	0·60, 1·00	0·78*	0·60, 1·00	0·78*	0·60, 1·00
≥ 4-year college	0·60***	0·46, 0·78	0·60***	0·46, 0·78	0·60***	0·46, 0·78
Race/ethnicity (ref=non-Hispanic White)						
Non-Hispanic Black	1·55**	1·14, 2·09	1·55**	1·14, 2·09	1·55**	1·14, 2·09
Hispanic	1·05	0·82, 1·36	1·05	0·82, 1·36	1·05	0·82, 1·36
Non-Hispanic Asian	0·60**	0·41, 0·89	0·60**	0·41, 0·89	0·60**	0·41, 0·89
Non-Hispanic Other	0·99	0·67, 1·47	0·99	0·67, 1·47	0·99	0·67, 1·47
Constant	0·53	0·23, 1·26	0·07***	0·03, 0·16	0·03***	0·02, 0·08

SSB, sugar-sweetened beverage.

* *P* < 0·05; **P < 0·01; ***P < 0·001.

† Natural feature includes ‘All natural’, ‘Organic’, ‘No HFCS’, ‘No artificial ingredients’ and ‘Non-GMO’.

Provision of other drink types was also associated with providing sweetened drinks. Caregivers who provided unsweetened juice/water blends were also more likely to report provision of sweetened drinks, as well as provision of sweetened milk drinks and other drink types. In contrast, provision of 100 % juice and serving plain water more than once a day were not associated with provision of sweetened drinks to their child. However, looking at the nutrition facts panel was not associated with providing sweetened drinks.

More-educated caregivers were less likely to provide sweetened drinks. Non-Hispanic Black caregivers were more likely to provide them (compared with non-Hispanic White caregivers), whereas non-Hispanic Asian caregivers were less likely to provide them. There were no differences between Hispanic and non-Hispanic White caregivers, and child’s age was not associated with providing sweetened drinks, after controlling for other factors.

The same independent variables were significant in the two models that compared frequency of providing sweetened drinks (never/low *v*. moderate/high and never-to-moderate *v*. high), with one exception. Reported importance of 100 % juice was not significantly related to frequency of sweetened drink provision in either model.

## Discussion

Consistent with previous studies on consumption of fruit drinks by young children^(3,20)^, most caregivers in our survey provided sweetened fruit-flavoured drinks (fruit drinks and/or flavoured water) to their child in the past month. One of every four caregivers provided them once a day or more often. Therefore, it is important to understand why caregivers provide sweetened drinks to their child despite child health experts’ guidance that young children should not consume drinks with added sugar or non-nutritive sweeteners^(3)^.

Socio-demographic differences in caregivers’ frequency of providing sweetened drinks after controlling for caregivers’ perceptions and behavioural factors were consistent with previous research on young children’s consumption of fruit drinks. Non-Hispanic Black caregivers provided sweetened drinks more frequently compared with non-Hispanic White caregivers^(20,21)^. The current study also included a large sample of non-Hispanic Asian caregivers and found that they provided sweetened drinks less frequently compared with other racial/ethnic groups.

These results also suggest that misperceptions about the healthfulness of sweetened fruit drinks likely contribute to frequent provision. As found in previous studies^(11,13)^, there was a positive relationship between caregivers’ perceptions of product healthfulness and frequency of provision, demonstrating the need to better inform caregivers that experts do not recommend serving fruit drinks and flavoured water to young children. In addition, the current study supports the importance of addressing common misperceptions about benefits of nutrition-related product features. Caregivers who believed that ‘Good source of vitamin C’ was important when choosing drinks for their child tended to provide sweetened drinks more frequently. On average 22 % of sweetened children drinks have this claim on the product package^(5)^, which indicates that caregivers may misunderstand the meaning of the vitamin C claim or believe that their child needs that nutrient. In contrast, caregivers who believed that 100 % juice and no/less sugar were important product features served sweetened drinks less frequently, indicating that raising awareness that sweetened drinks do not contain 100 % juice and do contain added sugar may be effective at reducing purchases.

Although popular children’s fruit drinks and flavoured water often contain non-nutritive sweeteners^(5)^, as well as added sugar, caregivers rated ‘no diet sweeteners’ as important features in the drinks they purchased for their child. This finding is consistent with previous research demonstrating that most parents are concerned about the safety of artificial sweeteners and do not want to provide products with non-nutritive sweeteners to young children^(11,22)^. However, caregivers in the current study also rated ‘low in energies’ (a common claim on products with non-nutritive sweeteners) as an important feature, and neither of these two factors were related to whether caregivers in our study served children’s fruit drinks and flavoured water to their child. Therefore, caregivers may not be aware that most children’s sweetened drinks contain diet sweeteners. In a previous study, approximately 60 % of caregivers could not accurately identify children’s fruit drinks and flavoured waters that contained non-nutritive sweeteners^(17)^.

In contrast, importance of other product features not related to nutrition was also associated with frequency of providing sweetened drinks. ‘My child asks for it’ was significantly related to providing these drinks more often, which indicates that marketing of these drinks directly to children likely has an impact on caregivers’ purchases by encouraging children’s ‘pester power’^(23)^. A previous study reported that companies spent $21 million to advertise fruit drinks and flavoured waters in 2018, and preschoolers (2–5 years old) and children (6–11 years old) saw more TV ads for these sweetened drinks than for drinks without added sweeteners in the same year. In addition, 90 % of these sweetened drinks contained child features on the packages^(5)^. The significant relationship between importance of the product coming in a box or pouch and frequency of providing these sweetened drinks also indicates that convenience and the ability to serve the products while on the go are important features in drinks they serve their children.

The current study also provides insights into some caregiver behaviours that may be associated with frequency of providing sweetened drinks to their child. Providing other sweetened drinks, including sweetened milk drinks, soda, diet soda and other SSB, was positively associated with frequency of providing fruit drinks and flavoured water, suggesting a pattern of serving multiple types of sweetened drinks in the household. However, despite recommendations that caregivers serve 100 % juice or water as healthier alternatives to sugary drinks^(3)^, the insignificant relationship between providing these drinks and frequency of caregivers’ providing sweetened drinks indicate that caregivers may not view them as substitutes for sweetened drinks. In addition, caregivers’ looking at the nutrition facts panel was not related to frequency of providing sweetened drinks, which was consistent with another study showing that viewing the panel did not affect caregivers’ choice of beverages^(24)^. It may be that consumers have difficulty interpreting the nutrition facts panel^(25)^, and even when shown the nutrition facts panel, many caregivers could not identify added sugar, juice and non-nutritive sweeteners in popular children’s drinks^(17)^.

The current study has some limitations. Respondents were recruited from an online panel of individuals who agreed to participate in online surveys and may not be representative of the entire population. In general, individuals who participate in online survey panels are more educated and have higher incomes than non-panel members^(26)^. Moreover, the sample was selected to allow for comparisons between racial/ethnic demographic groups. Compared with the overall population of US children under age 18, this sample included a higher proportion of Asian households (8 % *v*. 5 % for the population) and a lower proportion of Hispanic households (21 % *v*, 26 %), while the proportion of White and Black non-Hispanic households were comparable (50 % and 14 %, respectively)^(27)^. In addition, self-reported behaviours and attitudes may be subject to presentation and memory biases. Moreover, this cross-sectional survey cannot prove causal relationships. However, a study strength was the statistical model, which controlled for caregiver perceptions, purchasing/serving behaviours and demographic characteristics, to help explain how multiple individual factors are related to caregivers’ provision of sweetened drinks to their young children. Future research should also assess reasons for caregivers’ misperceptions about product features and reliance on marketing claims on product packaging and identify opportunities to address common misperceptions to reduce caregivers’ provision of sweetened drinks to their children.

These findings have implications for public health efforts to reduce consumption of sweetened drinks that contain added sugar and/or non-nutritive sweeteners by young children. Food companies should not advertise or use claims that imply benefits for children on products that do not meet expert recommendations for healthy children’s drinks. Health practitioners could provide clear guidelines to help identify healthy choices for children. Sugary drink taxes and restrictions on child-directed marketing of sweetened drinks would also help reduce consumption.

## Supplementary material

For supplementary material accompanying this paper visit https://doi.org/10.1017/S1368980022000751. click here to view supplementary material
