# Supplementary material for: Caregivers’ provision of sweetened fruit-flavoured drinks to young children: importance of perceived product attributes and differences by socio-demographic and behavioural characteristics
Source: Public Health Nutr. 2022 Apr 20;25(8):2308–16. doi: 10.1017/S1368980022000751 (PMC9991680; doi:10.1017/S1368980022000751)
Supplement: Supplementary file 1 [file S1368980022000751sup001.docx]

**Supplemental Table 1. Regression results with average marginal effects**

| Frequency of providing **sweetened** drinks to child | Never | | | Low  (Once a week or less) | | | Moderate  (A few times a week) | | | High  (Once a day or more) | | |
| --- | --- | --- | --- | --- | --- | --- | --- | --- | --- | --- | --- | --- |
|  | Coef |  | 95% CI | Coef |  | 95% CI | Coef |  | 95% CI | Coef |  | 95% CI |
| **Perceived healthfulness of sweetened drinks** | **0.000** | ^***^ | (-0.078, -0.050) | **-0.050** | ^***^ | (-0.068, -0.031) | **0.056** | ^***^ | (0.037, 0.076) | **0.058** | ^***^ | (0.043, 0.072) |
| **Importance of product features** |  |  |  |  |  |  |  |  |  |  |  |  |
| 100% juice | **0.027** | ^**^ | (0.010, 0.044) | -0.018 |  | (-0.038, 0.001) | -0.011 |  | (-0.029, 0.007) | 0.002 |  | (-0.018, 0.022) |
| Good source of vitamin C | **-0.013** | ^*^ | (-0.026, -0.001) | **-0.010** | ^*^ | (-0.018, -0.001) | **0.005** | ^*^ | (0.000, 0.011) | **0.018** | ^*^ | (0.002, 0.034) |
| No/less sugar | **0.018** | ^*^ | (0.002, 0.033) | **0.013** | ^*^ | (0.002, 0.024) | **-0.007** | ^*^ | (-0.014, -0.001) | -0.023 |  | (-0.043, -0.003) |
| No diet sweeteners | 0.005 |  | (-0.005, 0.014) | 0.003 |  | (-0.003, 0.010) | -0.002 |  | (-0.006, 0.002) | -0.006 |  | (-0.018, 0.006) |
| Low price | -0.006 |  | (-0.015, 0.003) | -0.004 |  | (-0.011, 0.002) | 0.002 |  | (-0.001, 0.006) | 0.008 |  | (-0.004, 0.019) |
| All natural, organic, no HFCS, no artificial ingredients, non-GMO | **0.020** | ^*^ | (0.003, 0.036) | **0.014** | ^*^ | (0.002, 0.026) | **-0.008** | ^*^ | (-0.015, -0.001) | **-0.026** | ^*^ | (-0.047, -0.004) |
| My child asks for it | **-0.015** | ^**^ | (-0.025, -0.006) | **-0.011** | ^**^ | (-0.018, -0.004) | **0.006** | ^**^ | (0.002, 0.010) | **0.020** | ^**^ | (0.008, 0.032) |
| Low in calories | -0.008 |  | (-0.017, 0.002) | -0.006 |  | (-0.012, 0.001) | 0.003 |  | (-0.001, 0.007) | 0.010 |  | (-0.002, 0.022) |
| Comes in juice box or pouch | **-0.008** | ^*^ | (-0.017, 0.000) | **-0.006** | ^*^ | (-0.012, 0.000) | 0.003 |  | (0.000, 0.007) | **0.011** | ^*^ | (0.000, 0.022) |
| **Freq of looking at nutrition facts panel**  (ref=never-sometimes) |  |  |  |  |  |  |  |  |  |  |  |  |
| Most of the time or all the time | -0.024 |  | (-0.056, 0.007) | -0.017 |  | (-0.040, 0.005) | 0.010 |  | (-0.003, 0.022) | 0.032 |  | (-0.009, 0.074) |
| **Served unsweetened juices in the past month** (ref=no) |  |  |  |  |  |  |  |  |  |  |  |  |
| 100% juice | -0.035 |  | (-0.107, 0.037) | -0.022 |  | (-0.059, 0.016) | 0.016 |  | (-0.019, 0.051) | 0.041 |  | (-0.034, 0.116) |
| Juice/water blends | **-0.058** | ^**^ | (-0.091, -0.025) | **-0.038** | ^***^ | (-0.059, -0.018) | **0.025** | ^**^ | (0.009, 0.040) | **0.072** | ^***^ | (0.034, 0.109) |
| **Served other sweetened drinks in the past month** (ref=no) |  |  |  |  |  |  |  |  |  |  |  |  |
| Sweetened milk drinks | **-0.087** | ^***^ | (-0.119, -0.056) | **-0.058** | ^***^ | (-0.078, -0.037) | **0.036** | ^***^ | (0.021, 0.051) | **0.109** | ^***^ | (0.072, 0.145) |
| Diet soda and other SSBs | **-0.155** | ^***^ | (-0.198, -0.111) | 0.028 |  | (-0.023, 0.080) | **0.064** | ^*^ | (0.013, 0.116) | **0.062** | ^*^ | (0.012, 0.112) |
| **Served plain water in the past month**  (ref=at most once a day) |  |  |  |  |  |  |  |  |  |  |  |  |
| More than once a day | 0.011 |  | (-0.017, 0.039) | 0.008 |  | (-0.013, 0.028) | -0.004 |  | (-0.016, 0.007) | -0.014 |  | (-0.052, 0.023) |
| **Age of 1- to 5-year-old child with the most recent birthday** | -0.010 |  | (-0.020, 0.001) | -0.007 |  | (-0.015, 0.001) | 0.004 |  | (0.000, 0.008) | 0.013 |  | (-0.001, 0.027) |
| **Caregiver's education** (ref= ≤ HS) |  |  |  |  |  |  |  |  |  |  |  |  |
| ≤ 2-year college | 0.037 |  | (0.000, 0.075) | **0.026** | ^*^ | (0.001, 0.051) | -0.015 |  | (-0.031, 0.001) | **-0.048** | ^*^ | (-0.094, -0.001) |
| ≥ 4-year college | **0.078** | ^***^ | (0.035, 0.122) | **0.050** | ^***^ | (0.025, 0.075) | **-0.034** | ^**^ | (-0.054, -0.013) | **-0.095** | ^***^ | (-0.142, -0.047) |
| **Race/ethnicity** (ref=non-Hispanic White) |  |  |  |  |  |  |  |  |  |  |  |  |
| Non-Hispanic Black | **-0.057** | ^**^ | (-0.092, -0.021) | **-0.049** | ^**^ | (-0.086, -0.013) | **0.017** | ^***^ | (0.009, 0.025) | **0.089** | ^**^ | (0.024, 0.154) |
| Hispanic | -0.008 |  | (-0.044, 0.029) | -0.006 |  | (-0.033, 0.022) | 0.003 |  | (-0.011, 0.017) | 0.010 |  | (-0.039, 0.060) |
| Non-Hispanic Asian | **0.084** | ^*^ | (0.012, 0.157) | **0.043** | ^**^ | (0.018, 0.067) | **-0.040** | ^*^ | (-0.078, -0.002) | **-0.087** | ^**^ | (-0.145, -0.028) |
| Non-Hispanic Other | 0.001 |  | (-0.055, 0.058) | 0.001 |  | (-0.039, 0.041) | -0.001 |  | (-0.024, 0.023) | -0.002 |  | (-0.076, 0.072) |

*** *p*<0.001, ** *p*<0.01, * *p*<0.05
